# Supplementary material for: Development of Saturated Fat Replacers: Conventional and Nano-Emulsions Stabilised by Lecithin and Hydroxylpropyl Methylcellulose
Source: Foods. 2022 Aug 22;11(16):2536. doi: 10.3390/foods11162536 (PMC9407586; doi:10.3390/foods11162536)
Supplement: Supplementary file 1 [file foods-11-02536-s001.zip › foods-1868633-supplementary.pdf]

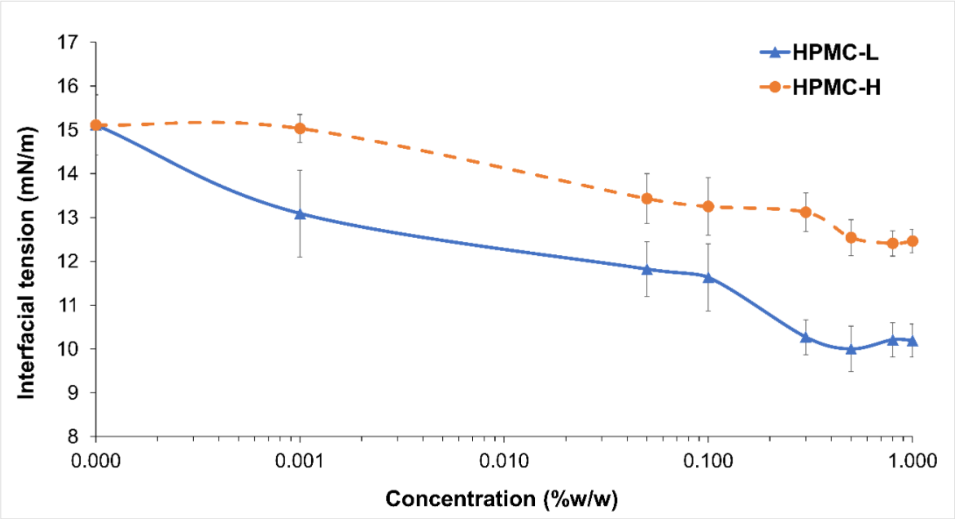

Figure S1. Characterisation interfacial tension of HPMC-L and HPMC-H.

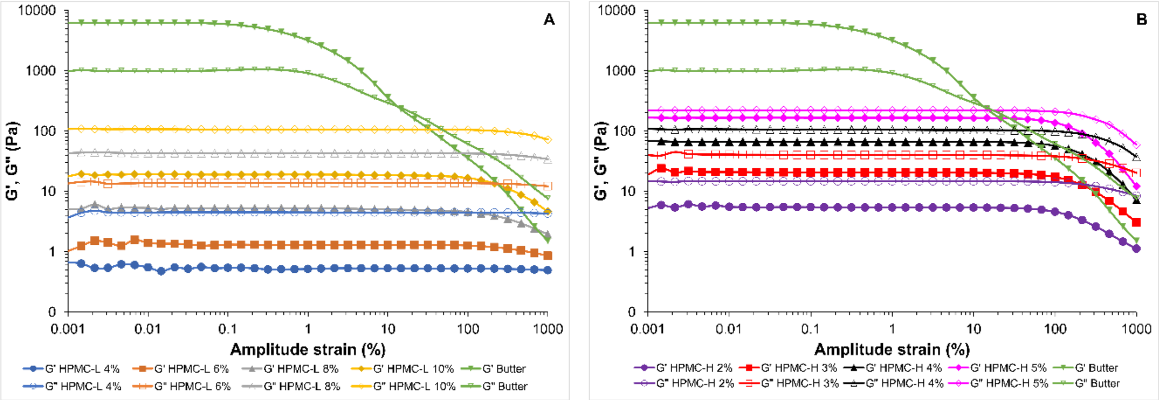

Figure S2. G' and G'' moduli of HPMC solutions and butter as a function of amplitude. A) HPMC-L solutions at different concentrations (4% blue circles; 6% orange squares; 8% grey triangles; 10% yellow diamond) and butter (green down-pointing triangle). B) HPMC-H solutions at different concentrations (2% purple circles; 3% red squares; 4% pink triangles; 5% black diamond) and butter (green down-pointing triangle). Filled symbols correspond to storage modulus (G') and open symbols to loss modulus (G'').

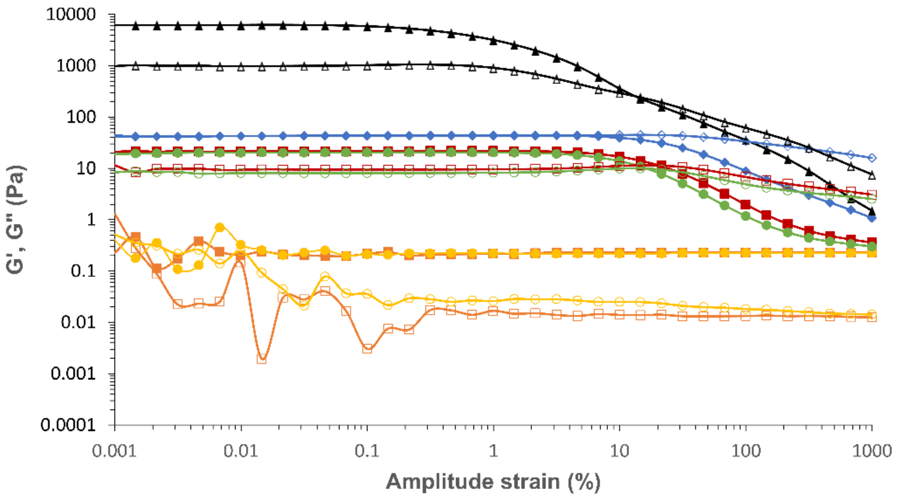

Figure S3. Dynamic moduli as a function of amplitude of emulsions and butter ▲. Conventional emulsion (CE-0 ■; CE-2 ■; CE-4 ◆). Nanoemulsion (NE-0 ●; NE-2 ●). Filled symbols correspond to elastic modulus (G') and open symbols to viscous modulus (G").

Table S1. MDD and PDI of conventional (CE) and nanoemulsion (NE) stabilised with lecithin and HMPC (0%, 2% and 4%).

Emulsions	MDD (nm)	PDI
CE-0	220.13[c] (2.22)	0.313[a] (0.030)
CE-2	257.51[b] (5.26)	0.356[a] (0.063)
CE-4	275.01[a] (6.78)	0.346[a] (0.035)
NE-0	185.83[e] (4.52)	0.230[b] (0.018)
NE-2	192.08[d] (3.93)	0.255[b] (0.013)

Indicated values are reported as means (standard deviation). Values with the different superscript letters are significantly different ($p < 0.05$) between samples in the same column.
